# Early high protein intake is associated with low mortality and energy overfeeding with high mortality in non-septic mechanically ventilated critically ill patients

**DOI:** 10.1186/s13054-014-0701-z

**Published:** 2014-12-14

**Authors:** Peter JM Weijs, Wilhelmus GPM Looijaard, Albertus Beishuizen, Armand RJ Girbes, Heleen M Oudemans-van Straaten

**Affiliations:** Department of Intensive Care Medicine, VU University Medical Center Amsterdam, De Boelelaan 1117, Amsterdam, The Netherlands; Department of Nutrition and Dietetics, Internal Medicine, VU University Medical Center Amsterdam, De Boelelaan 1117, 1081 HV Amsterdam, The Netherlands; Department of Nutrition and Dietetics, Amsterdam University of Applied Sciences, Dr. Meurerlaan 8 Amsterdam, The Netherlands; Institute for Cardiovascular Research, VU University Medical Center Amsterdam, De Boelelaan 1117, Amsterdam, The Netherlands; Department of Intensive Care Medicine, Medisch Spectrum Twente, Haaksbergerstraat 55, Enschede, The Netherlands

## Abstract

**Introduction:**

Early protein and energy feeding in critically ill patients is heavily debated and early protein feeding hardly studied.

**Methods:**

A prospective database with mixed medical-surgical critically ill patients with prolonged mechanical ventilation (>72 hours) and measured energy expenditure was used in this study. Logistic regression analysis was used to analyse the relation between admission day-4 protein intake group (with cutoffs 0.8, 1.0, and 1.2 g/kg), energy overfeeding (ratio energy intake/measured energy expenditure > 1.1), and admission diagnosis of sepsis with hospital mortality after adjustment for APACHE II (Acute Physiology and Chronic Health Evaluation II) score.

**Results:**

A total of 843 patients were included. Of these, 117 had sepsis. Of the 736 non-septic patients 307 were overfed. Mean day-4 protein intake was 1.0 g/kg pre-admission weight per day and hospital mortality was 36%. In the total cohort, day-4 protein intake group (odds ratio (OR) 0.85; 95% confidence interval (CI) 0.73 to 0.99; *P* = 0.047), energy overfeeding (OR 1.62; 95%CI 1.07 to 2.44; *P* = 0.022), and sepsis (OR 1.77; 95%CI 1.18 to 2.65; *P* = 0.005) were independent risk factors for mortality besides APACHE II score. In patients with sepsis or energy overfeeding, day-4 protein intake was not associated with mortality. For non-septic, non-overfed patients (n = 419), mortality decreased with higher protein intake group: 37% for <0.8 g/kg, 35% for 0.8 to 1.0 g/kg, 27% for 1.0 to 1.2 g/kg, and 19% for ≥1.2 g/kg (*P* = 0.033). For these, a protein intake level of ≥1.2 g/kg was significantly associated with lower mortality (OR 0.42, 95%CI 0.21 to 0.83, *P* = 0.013).

**Conclusions:**

In non-septic critically ill patients, early high protein intake was associated with lower mortality and early energy overfeeding with higher mortality. In septic patients early high protein intake had no beneficial effect on mortality.

**Electronic supplementary material:**

The online version of this article (doi:10.1186/s13054-014-0701-z) contains supplementary material, which is available to authorized users.

## Introduction

Optimal nutrition in terms of supplied energy and protein intake, in critically ill patients remains a topic of discussion. Especially, energy intake during the early phase of critical illness has been addressed. Several studies have shown that early low-energy (trophic) feeding does not influence survival and might even be beneficial [1-4]. However, in the optimal-energy groups of these studies, energy targets were calculated and not measured. As early energy-overfeeding may be harmful [5], it cannot be excluded that energy overfeeding contributes to worse outcome. Up to now, there have been no randomised studies investigating early protein-feeding per se. Observational studies have shown that protein intake according to current guidelines, 1.2 to 1.5 g/kg/day, is related to lower mortality [6-9]. Recent expert opinion even recommends more than 1.5 g/kg/day [10]. However, controversial results and hypotheses have been reported recently. In post-mortem muscle biopsies (12 patients), impaired autophagy correlated with the amount of infused amino acids [11]. Second, in a post-hoc analysis of the EPaNIC trial, the cumulative amount of protein/amino acid early during ICU stay was associated with delayed recovery [12]. Recently, a small observational study, including 50% patients with sepsis, reported a positive association between the change in muscle cross-sectional area in the first 1.5 weeks of ICU stay and protein intake, indicating more pronounced muscle wasting in the case of higher protein intake [13].

A proposed mechanism of the observed negative effect of protein is that early protein-feeding inhibits autophagy [11,14]. Autophagy provides a functional role in sepsis by promoting intracellular bacterial clearance [15]. Thus, early high-protein intake may especially be harmful in sepsis. In most studies, protein is provided in a fixed proportion to energy. We have been using several nutritional formulas with different protein/energy ratios and an algorithm to calculate both energy- and protein targets [16]. This allows us to study the effect of protein intake independent of energy intake. We previously found that mortality was lower in patients reaching both the energy and the protein targets, in contrast to energy targets alone. In that study, we analyzed the cumulative protein and energy provision over the entire period of mechanical ventilation, but in the present study we present new data on early protein- and energy-feeding [7].

The hypotheses underlying the present study are: 1) early protein intake of more than 1.2 g/kg according to ESPEN guidelines is beneficial [17]; 2) early high-protein intake could be harmful in patients with sepsis, possibly because of inhibition of autophagy; 3) early energy overfeeding is harmful and therefore might obscure the beneficial effect of early high-protein intake. To explore these hypotheses, we performed a post-hoc analysis with new prospective observational data on early (day 4) protein- and energy-intake and their association with hospital mortality, accounting for sepsis.

## Methods

This is a post-hoc analysis of new (unpublished) prospective observational data in a mixed medical-surgical ICU in a university hospital. Between August 2004 and March 2010, hemodynamically stable mechanically ventilated critically ill patients were included on days 3 to 5 when the predicted period of artificial nutrition was at least 5 to 7 additional days. Additional inclusion criteria were indirect calorimetry performed during ICU admission, age over 18 years, and first ICU admission. Exclusion criteria were inspired oxygen fraction (FiO_2_) >0.6, air leakage, and unavailable metabolic monitor data.

The study was approved by the ethics committee of the VU University Medical Center Amsterdam. Informed consent was waived because the study used variables routinely collected in clinical practice.

Early enteral nutrition (EN) was initiated in hemodynamically stable patients within the first 24 hours of ICU admission according to our protocol. The preferable route of administration was enteral. Parenteral nutrition (PN) was provided only when the gut failed (fistulas, short bowel, or obstruction) and was not given as parenteral supplementation to inadequate amounts of EN in the first week of nutritional therapy.

Energy requirements were initially calculated using the Harris and Benedict formula with an added 10% for activity and 20% for stress [7] and adjusted when indirect calorimetry was performed. Indirect calorimetry was performed using a Deltatracmonitor (Deltatrac™ MBM-100 Metabolic Monitor, Datex-Engstrom Division, Instrumentation Corp., Helsinki, Finland). Measurements were performed while patients were hemodynamically stable and calm, and ventilation allowed connection of the device. Enteral nutrition was deliberately continued during indirect calorimetry in order to assess total energy expenditure [18]. The new energy target was the measured energy expenditure with an added 10% for activity.

Protein was provided with a target of 1.2 to 1.5 g/kg pre-admission body weight. Protein intake was adjusted for body mass index (BMI) <20 kg/m^2^ to weight at BMI 20 kg/m^2^ and for BMI >30 kg/m^2^to weight at BMI 27.5 kg/m^2^ [19]. Protein provision was not reduced in case of renal failure, neither was it increased during continuous renal replacement therapy (CRRT).

The algorithm was used to determine the optimal nutritional formula and amount needed to meet both protein and energy requirements and accordingly indicated by the patient data management system (PDMS, Metavision®, IMD-soft, Tel-Aviv, Israel) in a dedicated spreadsheet [16]. The specific formulas were chosen for their different energy/protein ratios (Nutrison standard® and Nutrison Protein Plus®Nutricia, Zoetermeer, Netherlands), and Promote® (Abbott Nutrition, Abbott Park, IL, USA) [7].

Patient data, indirect calorimetry measurements, type of nutrition, pump settings, and protein and energy intake data from all sources including fluids and drugs, were hourly entered in the PDMS allowing accurate calculation of protein and energy intake. Protein and energy intake on day 4 was chosen as indicators of early intake, in line with the Dutch national nutritional performance indicator [20].

Energy intake on day 4 is expressed as the ratio of early (day 4) energy intake versus measured energy expenditure by indirect calorimetry. Energy overfeeding was defined by a ratio of >1.1 (yes/no). Protein provision on day 4 is expressed in g per kg pre-admission body weight. Allocation to septic and non-septic groups was based on the presence of severe sepsis or septic shock on ICU admission, using Surviving Sepsis Campaign guidelines criteria [21,22].

### Statistical analysis

Descriptive data are reported as mean and SD, median and interquartile range (for skewed distributions) or frequency and percentage. The Fisher exact test was used to compare categorical variables, and the chi square test was used to compare protein intake groups. Logistic regression analysis was performed with hospital mortality as the outcome variable and early protein intake group (with cutoffs of 0.8, 1.0, and 1.2 g/kg), early energy overfeeding (yes/no), and sepsis (yes/no) as independent variables, adjusted for acute physiology and chronic health evaluation (APACHE) II score. Separate analyses were performed for the cohorts with sepsis on ICU admission, with overfeeding, and finally non-septic non-overfed patients. Additionally a sensitivity analysis was performed with patients receiving at least 50% of measured energy expenditure. SPSS 20 (SPSS Inc., Chicago, IL, USA) was used for statistical analysis. A *P*-value <0.05 was considered statistically significant.

## Results

**Figure 1:**
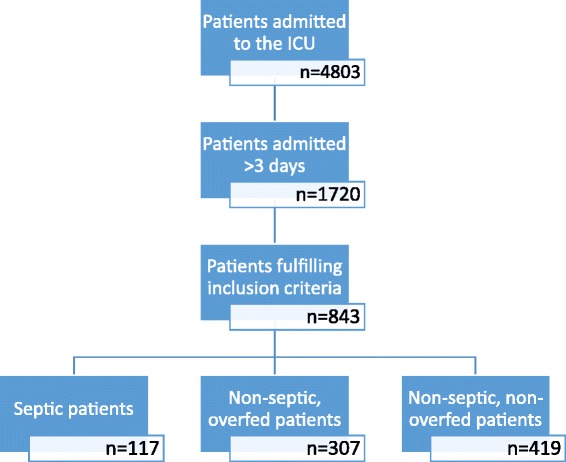
**Flow chart.**

During the study period, 4,803 patients were admitted to the ICU; 1,720 patients remained more than 3 days in the unit and 843 fulfilled the inclusion criteria (Figure 1). Nutrition during ICU stay was fully enteral in 618 (73%) and fully parenteral in 7 (1%) patients or mixed in 218 (26%) patients. Energy expenditure was measured median 5, mean of 7.2 ± 9.4 days after admission.

Overall (n = 843) mean energy intake at day 4 was 1,710 ± 699 kcal corresponding to 95% of measured energy expenditure (Table 1). Energy from sources other than nutrition (glucose and propofol) comprised median 132 kcal/day, accounting for 7.9% of the total energy intake; 10.1% in the non-overfed versus 6.4% in the overfed cohort (*P* <0.001).

**Table 1:** **Patient characteristics and outcome**

	**Septic patients**		**Non-septic overfed patients**		**Non-septic non-overfed patients**		**All patients**		**Analysis of variance, Kruskal-Wallis test or chi squared test,** ***P*** **-value**
	**n = 117**		**n = 307**		**n = 419**		**n = 843**		
	**Mean**	**SD**	**Mean**	**SD**	**Mean**	**SD**	**Mean**	**SD**	
Male gender, %	64.1		60.3		64.9		63.1		0.426
Age, y	64.2	14.2	61.3	17.1	63.2	16.4	62.6	16.4	0.172
Weight, kg	75.6^a,b^	20.3	74.2^a^	18.4	78.4^b^	16.7	76.5	18.0	**0.006**
Height, cm	172	10.7	172	9.7	173	9.6	172	9.8	0.804
Body mass index, kg/m2	25.4^a,b^	5.8	25.0^a^	5.8	26.3^b^	5.2	25.7	5.5	**0.006**
Body mass index <18.5, %	6.8		7.2		2.1		4.6		
Body mass index >30, %	12.8		12.7		18.5		15.3		
APACHE II score	25.4^a^	8.3	22.5^b^	7.5	17.9^b^	8.1	23.0	7.9	**0.001**
Respiratory rate, /minute^W^	22	6	21	7	21	7	21	7	0.188
VO_2_, ml/minute^W^	264^a,b^	69	254^a^	51	278^b^	60	268	60	**<0.001**
VCO_2_, ml/minute^W^	227^a,b^	56	224^a^	42	233^b^	51	229	49	**0.036**
Respiratory quotient^W^	0.87^a,b^	0.10	0.89^a^	0.11	0.84^b^	0.10	0.86	0.11	**<0.001**
FiO_2_, %^W, Z^	40	40 to 45	40	40 to 45	40	40 to 45	40	40 to 45	0.075
Measured EE^x^, kcal/d	1,808^a,b^	359	1,776^a^	318	1,886_b_	347	1,835	342	**<0.001**
Estimated EE^y^, kcal/d	1,991	415	1,983	365	2,049	368	2,017	375	**0.048**
Day 4 energy, kcal/d	1,673^a^	703	2,234^b^	430	1,337^c^	608	1,710	699	**<0.001**
Day 4 intake/measuredEE	0.95^a^	0.40	1.27^b^	0.18	0.71^c^	0.30	0.95	0.38	**<0.001**
Day 4 protein, g/kg	1.00^a^	0.53	1.33^b^	0.28	0.69^c^	0.43	0.97	0.49	**<0.001**
Length of ventilation^z^, d	18	12 to 29	16	10 to 28	17	10 to 28	17	10 to 28	0.495
Length of ICU stay^z^, d	22	14 to 34	19	12 to 31	20	12 to 31	20	12 to 31	0.516
Length of hospital stay^z^, d	32	21 to 55	37	22 to 59	35	21 to 59	35	21 to 59	0.544
ICU mortality, %	26.5		20.8		18.6		20.5		0.173
Hospital mortality, %	48.7		36.5		32.0		35.9		**0.004**

^a, b, c^Values in the same row not sharing the same letter are significantly different at *P* <0.05 in a post-hoc Bonferroni analysis or pairwise comparison on analysis of variance or Kruskal-Wallis test. ^W^Value at time of energy expenditure measurement. ^x^Energy target defined by indirect calorimetry. ^y^Energy target defined by Harris Benedict formula +30%. ^z^Non-normally distributed; data presented as median and 25^th^ to 75^th^ percentiles. *P*-values in bold indicate a significant test result. APACHE, acute physiology and chronic health evaluation; VO_2_, oxygen uptake; VCO_2_, carbon dioxide elimination; FiO_2_, inspired oxygen fraction; EE, energy expenditure.

Overall (n = 843) mean protein intake at day 4 was 0.97 ± 0.49 g/kg. When protein intake groups (<0.8, 0.8 to <1.0, 1.0 to <1.2, and ≥1.2 g/kg) were considered, and there was no difference in mortality; 37.6%, 35.4%, 35.4%, and 35.1% respectively, *P* = 0.930. To test the hypothesis that early protein intake has different effects in septic and non-septic patients, we analysed septic and non-septic patients separately.

### Septic patients

**Figure 2:**
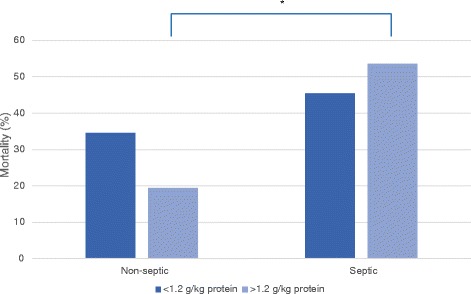
Hospital mortality for septic and non-septic patients with protein intake higher and lower than 1.2 g/kg. * *P* = 0.003.

The septic cohort consisted of 117 patients (14%) admitted with sepsis. Hospital mortality was significantly higher in septic patients than in non-septic patients (48.7% versus 33.9%, *P* = 0.003; Figure 2). The APACHE II score was higher as well (25.4 versus 22.6, *P* <0.001) (Table 1). Logistic regression analysis showed that mortality was not related to protein intake, energy overfeeding or APACHE II score in the septic cohort (Table 2).

**Table 2:** **Logistic regression analysis**

	**Odds ratio**	**95% CI**	***P*** **-value**
**All patients (n = 843)**			
Protein intake group^a^	0.85	0.73, 0.99	**0.047**
Energy overfeeding (yes/no)	1.62	1.07, 2.44	**0.022**
Sepsis (yes/no)	1.77	1.18, 2.65	**0.005**
APACHE II score	1.04	1.02, 1.05	**<0.001**
**Septic patients (n = 117)**			
Protein intake group^a^	1.15	0.80, 1.66	0.460
Energy overfeeding (yes/no)	0.82	0.35, 2.29	0.821
APACHE II score	1.03	0.98, 1.08	0.208
**Non-septic patients (n = 726)**			
Protein intake group^a^	0.80	0.67, 0.95	**0.011**
Energy overfeeding (yes/no)	1.89	1.19, 3.02	**0.007**
APACHE II score	1.04	1.01, 1.06	**0.001**
**Non-septic overfed patients (n = 307)**			
Protein intake group^a^	0.91	0.59, 1.40	0.666
APACHE II score	1.04	1.00, 1.07	**0.029**
**Non-septic non-overfed patients (n = 419)**			
Protein intake group^a^	0.77	0.63, 0.93	**0.008**
APACHE II score	1.03	1.01, 1.06	**0.013**

^a^Protein intake groups were <0.8, 0.8 to <1.0, 1.0 to <1.2, and ≥1.2 g/kg. *P*-values in bold indicate a significant test result. APACHE, acute physiology and chronic health evaluation.

### Non-septic overfed and non-overfed patients

**Figure 3:**
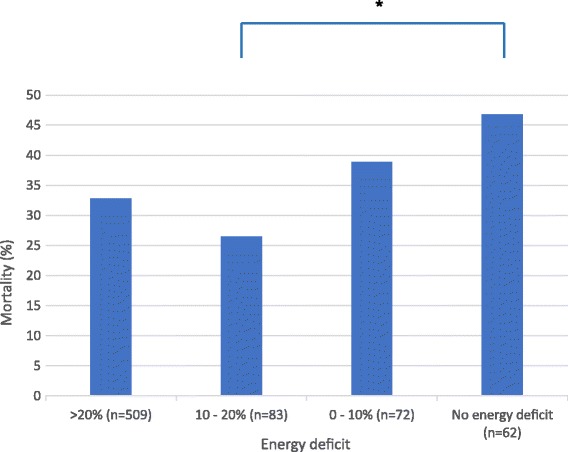
**Hospital mortality for cumulative energy deficit over the first 4 days of ICU stay for non-septic patients (n = 726;** ***P*** **= 0.053).** Reference is the measured resting energy expenditure of the patient. *P = 0.012.

Patient characteristics and nutritional data of non-septic overfed patients (n = 307) and non-septic non-overfed patients (n = 419) are shown in Table 1. In the non-septic cohort hospital mortality was not significantly higher in the day-4 overfed patients than in the non-overfed group (36.4% versus 32.1%, *P* = 0.234), the APACHE II scores were similar and energy intake in the non-overfed group was only 71% of measured EE. Figure 3 shows the cumulative energy deficit over the first 4 days of ICU stay (n = 726), with worst hospital mortality outcome in the overfed group (*P* = 0.053).

In this non-septic cohort (n = 726), logistic regression analysis demonstrated that the day-4 protein intake group (odds ratio (OR) = 0.80, 95% CI 0.67, 0.95, *P* = 0.011), day 4 overfeeding (OR = 1.89, 95% CI 1.19, 3.02, *P* = 0.007), and APACHE II score (OR = 1.04, 95% CI 1.01, 1.06, *P* = 0.001) had significant independent impact on mortality (Table 2). Thus, high day-4 protein intake was related to lower mortality in non-septic patients, while day-4 overfeeding and higher APACHE II score were related to higher mortality. The day-4 protein intake group was not related to mortality in the non-septic overfed group (Table 2).

### Non-septic and non-overfed patients

**Figure 4:**
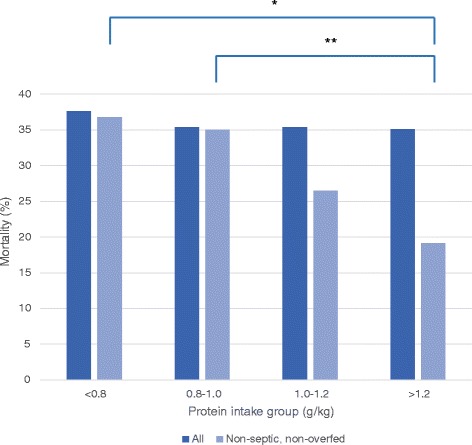
**Hospital mortality for all patients per protein intake group and for all non-septic and non-overfed patients per protein intake group.** *P = 0.008; **P = 0.047.

In patients who were not septic and not overfed (n = 419), the higher protein intake group was associated with lower mortality (Table 3). Hospital mortality was 36.8%, 35.0%, 26.5%, and 19.1% for the <0.8, 0.8 to <1.0, 1.0 to- <1.2, and ≥1.2 g/kg protein-intake groups respectively (*P* = 0.033). Hospital mortality was 34.5% for day-4 protein intake <1.2 g/kg versus 19.1% for day-4 protein intake ≥1.2 g/kg (*P* = 0.015; Figure 4). Regression analysis with dummies for protein intake groups showed that the effect of protein was only significant at a protein intake level of ≥1.2 g/kg (OR = 0.42, 95% CI 0.21, 0.83, *P* = 0.013).

**Table 3:** **Patient characteristics and outcome in non-septic, non-overfed patients**

	**<0.8 g/kg**		**0.8 to <1.0 g/kg**		**1.0 to <1.2 g/kg**		**≥1.2 g/kg**		**All non-septic, non-overfed patients**		**Analysis of variance, Kruskal-Wallis test or chi squared test,** ***P*** **-value**
	**n = 223**		**n = 60**		**n = 68**		**n = 68**		**n = 419**		
	**Mean**	**SD**	**Mean**	**SD**	**Mean**	**SD**	**Mean**	**SD**	**Mean**	**SD**	
Male gender, %	66.4		60.0		67.6		61.8		64.9		0.714
Age, y	63.9	16.5	61.6	16.2	62.7	15.6	62.7	17.2	63.2	16.4	0.775
Weight, kg	78.5	17.5	79.6	15.1	80.4	17.3	75.1	14.7	78.4	16.7	0.278
Height, cm	173	9.5	173	9.2	173	9.7	171	10.5	173	9.6	0.556
Body mass index, kg/m2	26.2	5.3	26.9	5.6	26.8	5.5	25.6	3.7	26.3	5.2	0.439
Body mass index <18.5, %	2.7		1.7		0.0		2.9		2.1		
Body mass index >30, %	17.9		21.7		17.6		14.7		17.9		
APACHE II score	22.7	8.4	22.2	7.8	22.4	7.6	22.9	7.5	22.6	8.1	0.961
Respiratory rate, /minute^W^	20	6	19	6	21	5	22	8	21	7	0.059
VO_2_, ml/minute^W^	274	64	270	51	286	58	292	55	278	60	0.080
VCO_2_, ml/minute^W^	228^a^	54	226_a_	40	239^a,b^	43	251^b^	53	233	51	**0.005**
Respiratory quotient^W^	0.84	0.10	0.85	0.12	0.85	0.10	0.86	0.08	0.84	0.10	0.436
FiO_2_, %^W, Z^	40	40 to 45	40	40 to 45	40	40 to 45	40	40 to 45	40	40 to 45	0.460
Measured EE^x^, kcal/d	1,860	367	1,864	299	1,953	310	1,924	348	1,886	347	0.181
Estimated EE^y^, kcal/d	2,049	378	2,066	333	2,088	365	1,993	371	2,049	368	0.478
Day-4 energy, kcal/d	904^a^	453	1,679^b,c^	256	1,840^c,d^	283	1,950^d^	353	1,337	608	**<0.001**
Day-4 intake/measuredEE	0.49^a^	0.25	0.91^b,c^	0.09	0.95^c,d^	0.10	1.02^d^	0.09	0.71	0.30	**<0.001**
Day-4 protein, g/kg	0.35^a^	0.24	0.86^b^	0.06	1.05^c^	0.05	1.30^d^	0.10	0.69	0.43	**<0.001**
Length of ventilation^z^, d	17	11 to 29	20	11 to 28	15	8 to 27	17	9 to 24	17	10 to 28	0.128
Length of ICU stay^z^, d	20	13 to 32	22	13 to 31	17	9 to 29	19	10 to 29	19	12 to 31	0.178
Length of hospital stay^z^, d	35	19 to 60	40	26 to 63	30	19 to 61	34	23 to 49	35	21 to 59	0.447
ICU mortality, %	22.0		18.3		17.6		8.8		18.6		0.111
Hospital mortality, %	36.8		35.0		26.5		19.1		32.0		**0.033**

^a, b, c, d^Values in the same row not sharing the same letter are significantly different at *P* <0.05 in a post-hoc Bonferroni analysis or pairwise comparison in the analysis of variance or Kruskal-Wallis test. ^W^Value at time of energy expenditure measurement. ^x^Energy target defined by indirect calorimetry. ^y^Energy target defined by Harris Benedict formula +30%. ^z^Non-normally distributed; data presented as median and 25^th^ to 75^th^ percentiles. P-values in bold indicate a significant test result. APACHE, acute physiology and chronic health evaluation; VO_2_, oxygen uptake; VCO_2_, carbon dioxide elimination; FiO_2_, inspired oxygen fraction; EE, energy expenditure.

Adjustment for patients with any use of parenteral nutrition did not change the results. BMI was not a significant predictor of mortality either in the whole group or in subgroup analysis.

### Possible underfeeding effect

To further explore whether the higher mortality in the low protein-intake group in non-septic non-overfed patients was caused by energy underfeeding rather than low protein feeding, a sensitivity analysis on energy intake was performed. In the <0.8 g/kg protein group, 108 out of 223 patients were seriously underfed (defined as <50% of measured energy expenditure (EE)). Mortality was 37.2% including all patients in the <0.8 g/kg protein group, and 40.8% excluding the patients with <0.8 g/kg protein and an energy intake of <50% of measured EE. Thus, the contrast between the <0.8 g/kg group and the >1.2 g/kg group increased when the seriously underfed group in terms of EE was excluded (40.8% versus 19.4%, *P* = 0.012). In the ≥1.2 g/kg-protein group none of the patients were energy underfed. When comparing the ≥1.2 g/kg-protein group to the <0.8 g/kg-protein group, the OR for mortality for the ≥1.2 g/kg-protein group was 0.38 (95% CI 0.18, 0.81) in those receiving >50% of EE, and 0.22 (95% CI 0.06, 0.77) in those receiving optimal energy intake (90-100% of EE).

## Discussion

This post-hoc observational study in critically ill patients undergoing prolonged mechanical ventilation shows that high early-protein intake (defined as intake at day 4) is associated with lower hospital mortality and early energy overfeeding with higher mortality, independent of APACHE II score and the presence of sepsis. A benefit of early high-protein intake was only found in the non-septic and non-overfed patients and not in patients admitted with sepsis and in those with early energy overfeeding. The lowest mortality was found in non-septic patients without overfeeding receiving >1.2 g/kg protein (pre-admission weight). Thus, our findings justify the current recommendation on protein intake in patients without sepsis being at least 1.2 g/kg as early as day 4 of ICU admission [17]. They also stress the importance of measuring EE to prevent early overfeeding. However, for septic patients a clear protein recommendation cannot be given based on this study.

### Protein

Up to now, early protein feeding per se has not been evaluated in randomised studies. In the previous analysis of this patient cohort we showed that a protein intake of more than 1.2 g/kg over the whole period of mechanical ventilation was associated with lower mortality [6,7]. Reaching energy targets alone was not sufficient. These results were confirmed and extended by the observational study of Allingstrup *et al*., showing a decrease in mortality with increasing protein intake up to 1.5 g/kg [8].

A recent secondary analysis of an observational study including 2,270 septic ventilated medical patients receiving enteral nutrition with a mean of 14.5 kcal/kg and 0.7 g/kg protein per day found that both an increase of 1,000 kcal and of 30 g protein per day, thus a delivery closer to recommended protein intake, was associated with reduced mortality [9]. This study covered the mean energy and protein intake for a maximum of 12 days or until death after ICU discharge, and did not address intake as early as day 4. Of note, mean protein and energy intakes in this septic cohort were lower than in our (septic) cohort, and protein and energy intake were related.

However, results of randomised studies are confusing, because early underfeeding, which implies low protein intake, appears not to increase mortality [1,4]. Supplemental PN was even associated with an increased infection rate and higher duration of mechanical ventilation and renal replacement therapy in the largest trial [1]. An explanation could be that nutrition inhibits autophagy [23]. Autophagy is considered a housekeeping system to remove dysfunctional and toxic proteins and complete cellular structures [11,23], and the degradation products subsequently provide nutritional substrate. In a sub-study of the EPanic trial, late PN was associated with reduced muscle weakness and more efficient autophagic control of muscle fibres [24], although the final muscle weakness assessment was not different. Which nutritional component contributes to inhibition of autophagy most is as yet unknown. However, some studies suggest that protein might be more important than glucose [11,12], although Casaer *et al*. did not adjust for energy intake [12]. This suggestion seems to contradict our finding that early high-protein intake was associated with lower mortality. However, our study also shows that in the septic cohort early high-protein intake was not associated with lower mortality, which is in line with the patient group of Puthucheary *et al*. [13] of which half was admitted with sepsis. We hypothesized that septic patients could behave differently because autophagy is also used to degrade intracellular micro-organisms [25]. Thus, autophagy provides a functional role in sepsis by promoting intracellular microbial clearance. Furthermore, in a recent study in critically ill septic patients receiving PN, muscle protein synthesis was normal, but protein breakdown was increased up to 260% compared to healthy controls [26]. Apparently, feeding could not suppress increased protein breakdown in these septic patients. In favour of protein are the randomised trials on supplemental parenteral nutrition that administered a higher amount of protein [2,27], measured energy expenditure [27], and found a positive impact of supplemental parenteral nutrition on clinical outcomes [2,27]. Taken together there appears to be a delicate balance in the critically ill patient, and timing and dosing of protein and energy in specific disease groups will have to be addressed in future randomised studies.

### Overfeeding

Our study also showed that day-4 energy overfeeding was harmful. Overfeeding was defined as an energy intake of more than 110% of measured EE. Forty-one percent of our patients appeared to be overfed on day 4. This means that a standard prescription of estimated energy requirement by Harris-Benedict equation plus 30% is an inaccurate predictor of energy requirements in ICU patients, as has been reported before [5,28,29]. Although newer formulas might be more accurate [30], measurement of EE by indirect calorimetry remains the most appropriate tool. However, even when knowing actual EE, it is not known whether energy supply should cover the full equivalent of energy expenditure during the first days of critical illness, because nutrition cannot suppress early endogenous glucose production which may provide more than 50% of energy expenditure [5]. Figure 3 suggests that mild (10 to 20%) underfeeding of energy in the early period of ICU stay might be beneficial. Of note, the increased rate of infections and longer duration of mechanical ventilation and renal replacement therapy found in the EPaNIC trial [1] could partially be explained by a component of overfeeding, because energy needs were not measured but calculated, and rather high energy targets were attained. A smaller study applying calorimetry-tailored nutrition found a trend to lower mortality rate in the supplemental PN group, but also an increased infection rate and longer ventilation and ICU stay [31]. Remarkably, both energy and protein intake were higher in the calorimetry-tailored group. Furthermore, the Swiss trial observed fewer infections in the group receiving supplemental PN from day 4 [27]. Energy intake was tailored by calorimetry and protein intake was higher than in the EPaNIC study. Finally, in the Australian trial, supplemental PN from day 1 provided no effect on mortality or infection rate, but decreased muscle wasting, ventilator duration, and improved quality of life [2]. Thus, in the last three trials, a higher protein and energy intake was beneficial. Whether in favour of targeted feeding or not, these studies were not designed to investigate early protein-targeted feeding and in some of these studies some of the patients were likely overfed [32].

Our study has several limitations, in particular its observational design. A lower early-protein intake may reflect a higher severity of disease. However, protein intake remained a predictor of mortality, independent of APACHE II score, the standard estimate of mortality in critically ill patients. Also, an improved energy intake but with insufficient protein (0.8 to 1.2 g/kg) was not associated with lower mortality. We previously showed that the application of our nutritional algorithm improved adequate protein supply at day 4 from about 30% to almost 60%. However, despite our algorithm, not all patients received adequate protein intake. Furthermore, estimating EE using the old Harris-Benedict equation +30% appears to be associated with significant overfeeding. In addition, the measurement of EE was generally measured after day 4. This means that overfeeding could have been ongoing for a couple of days before it was noticed and corrected. Unfortunately, in clinical practice daily measurement of EE is not feasible. Finally, the number of septic patients was relatively low, but proportional to the admission pattern of the unit.

A strong point of our study is the distinction between patients with and without early energy overfeeding. The negative effect of early overfeeding on patient outcome supports the notion that measuring EE is crucial for optimizing nutrition. Another strong point of this study is that the sample size was large enough to independently assess the effect of protein intake in patients with and without sepsis. Finally, EE was measured while feeding was continued, thereby reflecting real-life total EE.

## Conclusion

The present post-hoc analysis of a prospective observational study shows that early protein intake at a level of ≥1.2 g/kg at day 4 of ICU admission is associated with lower and early energy overfeeding with higher hospital mortality in critically ill patients with prolonged mechanical ventilation without sepsis. The possible benefit of early high-protein feeding should be confirmed by a randomised controlled trial.

## Key messages

- Early protein intake at a level of ≥1.2 g/kg at day 4 of ICU admission is associated with lower and early energy overfeeding with higher hospital mortality in non-septic mechanically ventilated critically ill patients.
- In patients with sepsis at admission, no relation was found between early protein intake and mortality.
- In patients with early energy overfeeding, no relation was found between early protein intake and mortality.

## Abbreviations

APACHE: acute physiology and chronic health evaluation
BMI: body mass index
CRRT: continuous renal replacement therapy
EE: energy expenditure
EN: early enteral nutrition
FiO_2_: inspired oxygen fraction
OR: odds ratio
PN: parenteral nutrition
VCO_2_: carbon dioxide elimination
VO_2_: oxygen uptake
